# Developing and Integrating Advanced Movement Features Improves Automated Classification of Ciliate Species

**DOI:** 10.1371/journal.pone.0145345

**Published:** 2015-12-17

**Authors:** Ali Soleymani, Frank Pennekamp, Owen L. Petchey, Robert Weibel

**Affiliations:** 1 Department of Geography, University of Zurich, Zurich, Switzerland; 2 Institute of Evolutionary Biology and Environmental Studies, University of Zurich, Zurich, Switzerland; 3 Eawag: Swiss Federal Institute of Aquatic Science and Technology, Department of Aquatic Ecology, Dübendorf, Switzerland; Universitat de Valencia, SPAIN

## Abstract

Recent advances in tracking technologies such as GPS or video tracking systems describe the movement paths of individuals in unprecedented details and are increasingly used in different fields, including ecology. However, extracting information from raw movement data requires advanced analysis techniques, for instance to infer behaviors expressed during a certain period of the recorded trajectory, or gender or species identity in case data is obtained from remote tracking. In this paper, we address how different movement features affect the ability to automatically classify the species identity, using a dataset of unicellular microbes (i.e., ciliates). Previously, morphological attributes and simple movement metrics, such as speed, were used for classifying ciliate species. Here, we demonstrate that adding advanced movement features, in particular such based on discrete wavelet transform, to morphological features can improve classification. These results may have practical applications in automated monitoring of waste water facilities as well as environmental monitoring of aquatic systems.

## Introduction

Over the past decades, various tracking technologies such as the Global Positioning System (GPS) and sophisticated video techniques have become accessible to scientists and enabled the recording of large amounts of data about the movement paths of individual organisms [1–4]. GPS tags or collars have the advantage that auxiliary information on the individual can be collected when the device is attached, which can subsequently help in understanding the differences between collected movement paths. Typically the target of these movement analyses is to infer movement patterns corresponding to behaviors such as foraging or dispersal [5,6] or to link the genotype and behavioral phenotype of organisms [7]. However for inferring other sorts of information such as gender or species, remote techniques such as video tracking are neither capturing nor marking the individual and hence auxiliary information on the species or gender of the tracked individual is not known. Previous studies found that it is possible to distinguish trajectories based on individual features such as their genotype and gender [7], the degree of light availability [8] or whether individuals were in resource poor or rich environments [9]. Moreover, individual movement may also be indicative of the internal state of the moving individual, which can be used to evaluate the effect of toxic substances in the environment or laboratory based toxicity assays [10]. Developing techniques to infer behavior from movement paths is an active field of research [11–14] especially for GPS-based movement data, but here we focus on classifying trajectories regarding species identity. This problem is less well studied as remote tracking studies, where multiple species (or genotypes) interact, are still relatively rare in ecology, but are expected to increase rapidly with high-throughput analysis based on image and video analysis [3]. Regardless of the tracking technique used, all these applications have in common that characteristic features of the movement have to be associated to known classes, such as behavior or species identity, which is generally referred to as *movement classification*.

Movement classification represents a particular set of problems, where either entire movement paths (trajectories) or parts of trajectories (i.e. subtrajectories) are assigned to classes with respect to some *a priori* unknown criterion. As in all classification problems, training in which the class is known is used to infer criteria (characteristic features of the data) that are able to reliably predict the class of unknown cases. Here, we address how different features of the movement data contribute to classification accuracy.

In particular, we examine how movement data can contribute to classifying different species of ciliates (*Kingdom Protozoa*, *Alveolata*, *Ciliophora*). Ciliates are widely found in all types of fresh-water and marine environments and fulfill important functions in natural ecosystems such as controlling the abundance of bacteria by predation and are themselves important food for small invertebrates such as crustaceans (e.g. Daphnia water fleas) [15,16]. Ciliates are also widely used as model organisms in studies in ecology and evolutionary biology where experimental microcosms (i.e. small-sized standardized containers with tight environmental control) are used [17]. Only recently due to the advent of automated video analysis, quantitative traits such as movement (e.g. speed, linearity) and morphology (e.g. cell size, cell shape) can be measured on large numbers of individuals automatically and hence are explicitly considered in such microcosm studies [17].

Morphological attributes are commonly used to classify ciliate species [18–21]. Our goal here is to investigate how movement of ciliates can contribute to their classification, as well as the performance of movement features only in the classification. We make this distinction to draw general conclusions accounting for cases where information on morphology is missing and only movement features as classification inputs are available. Microbial species are often characterized by little morphological differentiation, even though they are known to be physiologically and genetically diverse [22]. Hence, movement behavior may be a better indicator of taxonomy than morphology, or at least assist with morphological based classification. Automated video based classification of ciliate species has potential application in different fields, for instance for the automated monitoring of waste water facilities as well as environmental monitoring of aquatic systems more broadly [21].

Whereas previous analysis of the data has shown that movement can improve classification [23], here we aim to systematically explore the contributions of more sophisticated movement analysis techniques to classification. Feature extraction from movement data is complicated by two characteristics of movement. First, considering that movement operates through space and time, representing and integrating both of these domains remains a challenge [24]. Respectively in movement classification, relevant features in the spatial and temporal domains should be extracted in order to capture spatiotemporal (as opposed to separate spatial, or temporal) characteristics of the moving individual under study. Second, the patterns underlying the movement classes might relate to multiple spatial and temporal scales (i.e. instantaneous, diurnal or seasonal) and using only the original temporal granularity for calculating MPs is a strong oversimplification of actual movement patterns [25]. Thus, distinguishing features may only become apparent if multiple analysis scales are considered [26].

In this study, wavelet analysis is investigated as a cross-scale analysis approach for extracting features in movement classification. While the related technique of Fourier transform is helpful for identifying periodicities in stationary time series, it will fail on time series where periodicity occurs only irregularly through the data set [27,28]. This is the case for most movement time series, as these are often non-homogeneous, made up of a combination of discrete behaviors. For example, animals may spend more time in a nesting or resting place and thus show only limited movement [29]. In contrast, other places may be used intermittently for foraging and animals may show more movements and hence higher activity [30]. Hence, we test whether integrating features based on wavelet analysis could improve classification due to its ability to detect non-stationary patterns in movement data, where transient types of activity occur. Moreover, features based on the wavelet transform can also be useful for relating these activities to other factors (e.g. physiological, ecological, contextual, etc.) affecting movement [31,32]. Thus, features based on wavelet transform are considered as a complementary tool for identifying the elements of periodic patterns in the movement data.

The contributions of this paper are two-fold. First, we develop a model for movement classification purely based on quantitative features, where each feature measures particular aspects of movement. Three sets of movement features are used (movement parameters only, approximate entropy (ApEn), and wavelet coefficients) and compared to the baseline model that uses only morphological features. We show how gradually adding features improves the performance of the classification model. Secondly, we demonstrate that careful selection and integration of movement features will lead us to comparable results, irrespective of the classification method employed, i.e. decision trees (DT) vs. support vector machines (SVM). Although the results of the classification method might differ among the individual sets of features used, once all features are integrated, the obtained results are comparable between the two classification methods.

## Methods and Materials

### Overview of movement classification

**Fig 1:**
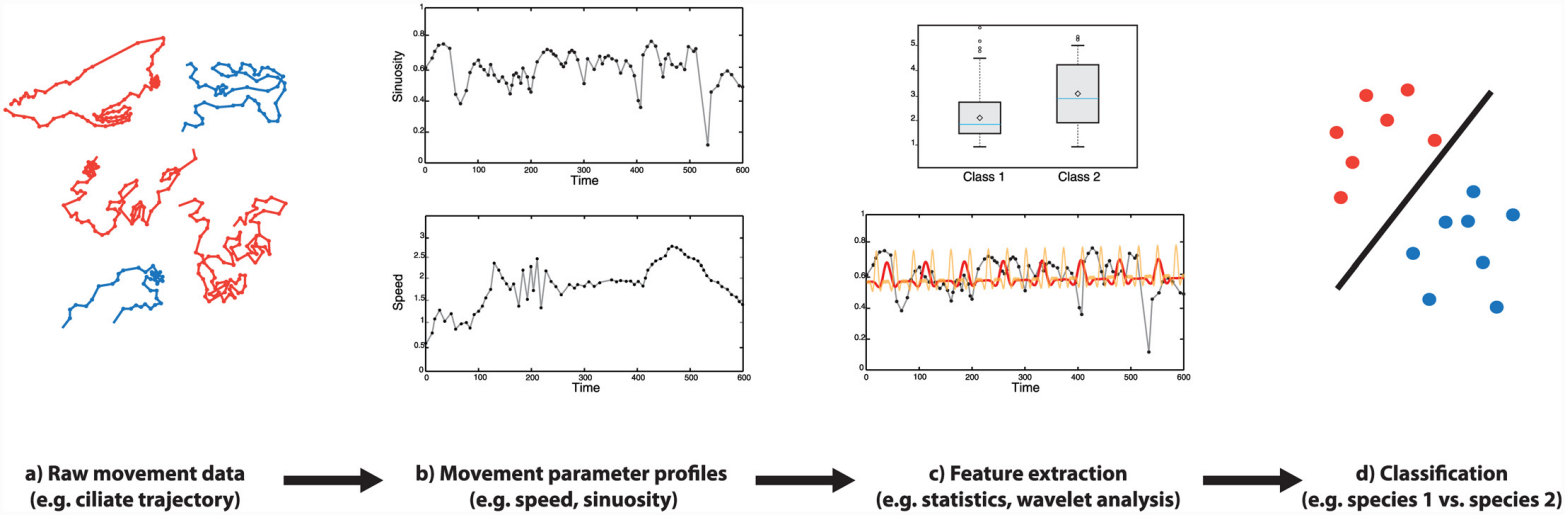
Overview of the movement classification process. a) raw trajectories of two species of ciliates consisting of time-stamped X- and Y coordinates; b) movement parameters are calculated from the locations and MP profiles through time are obtained; c) extraction of features, for instance, summary statistics of movement parameters (upper panel) or wavelet coefficients (lower panel); d) classification of species based the movement features extracted.

As in any general classification problem, several steps need to be taken in movement classification in order to make the transition from the observational movement data to the final classes, which we have schematized in Fig 1. The movement parameters (MP, e.g. speed, acceleration, turning angle, etc. [33]) are calculated from the raw movement data. Since trajectories are ordered by time, we get a time series of MP values, which we call an MP profile. The obtained MP profiles are converted to a set of feature vectors, on which statistical descriptors (i.e. mean, standard deviation, median, etc.) may be computed. In this study, we use approximate entropy and discrete wavelet transform to provide additional features. The classification model is built by using the relevant extracted features as quantitative inputs for the model and relating these to the known classes.

#### Extraction of movement features

Seven movement parameters (i.e. distance travelled, speed, acceleration, turning angle, angular velocity, meandering and sinuosity) were calculated. These values were aggregated into concise representations (i.e. features) to be used in the classification. Features can be related to individual fixes [25], to a short series of fixes, for instance by segmenting the trajectories [34], or to all fixes in a recorded trajectory [26]. Here, we consider two categories of features: aggregate features computed on the whole trajectories and features based on the wavelet transform.

#### Aggregate movement features

Moment statistics of movement parameters are the most common form of aggregate features used in classification models. By describing general variations present in the movement data, these features may already differentiate between movement classes at a certain scale. Different moment statistics may be used, such as minimum, maximum, median, mean, standard deviation, etc. However, in the transition from the raw movement trajectories to the summarized representation of classification features, an information loss will be introduced: by the use of only aggregated features at a certain scale, clearly not all aspects of movement can be detected [26].

Therefore, we also used ApEn values as an added feature in the classification model [35,36]. ApEn is a method from time series analysis for quantifying regularities and fluctuations in sequential data [35]. Since moment statistics might ignore subtle changes in the structure of MP profiles, ApEn values are calculated to investigate the regularity or to detect dominant fluctuations in such profiles. As a measure of system complexity, higher values of ApEn suggest a more random distribution (i.e. less predictable profile with complex structure), while a smaller value implies less complexity and more regularity (i.e. highly structural profiles containing many repetitive patterns). In order to better distinguish between movement classes, approximate entropy of MP profiles can be used to show how the structural complexity of particular movement parameters varies over time [36].

#### Feature extraction based on wavelet analysis

Based on the MP profiles, the discrete wavelet transform (DWT) was used in order to decompose the movement signal into different levels (see S1 Text, S1 and S2 Figs for a detailed description of the wavelet transform performed). Wavelet analysis can reveal features such as temporal autocorrelation or periodicity patterns in the movement data [37,38], which may go undetected through the aggregate features mentioned before. In each decomposition level, an approximation and a detail sub-band is obtained, yielding two sets of wavelet coefficients. These two sets of information are sufficient to reconstruct the signal [39,40]. Three moment statistics of wavelet coefficients in each sub-band were considered as input features. These include the mean of the absolute values of the coefficients in each sub-band; average power of the wavelet coefficients in each sub-band; and standard deviation of the coefficients in each sub-band. There are two parameters to be set in a DWT analysis: the first is the choice of mother wavelet function, through which the signal is passed in order to characterize the variations. All the wavelets used at different levels of decompositions are scaled and shifted versions of the same mother wavelet function. A Daubechies wavelet (db4) was chosen as the mother wavelet function, due to its superior performance, and order 4 selected to detect the discontinuities in the signals [30,41]. The second parameter is the number of the decomposition levels to provide approximation and detail sub-band at different scales. Considering that possible decomposition levels depend on the length of the trajectories, this was chosen to be 5 in this study.

Other studies have used the distance travelled and speed in the wavelet analysis [30,31,38]. For both, periodic patterns in the profile may be expected whereas for other parameters, it would be rather difficult to interpret the periodicity occurring in the profiles. In the experiments reported here, we used the profiles of the distance travelled to extract the wavelet-based features.

## Experiment

### Model species

We used 8 species of small, single-celled ciliates as model species for this study: *Paramecium caudatum*, *Paramecium aurelia*, *Blepharisma japonicum*, *Colpidium striatum*, *Colpidium campylum*, *Cyclidium glaucoma*, *Tetrahymena thermophila* and *Loxocephalus* sp.

Each ciliate stock was cultured separately in a jar of 240 ml volume covered by aluminum cover to allow air exchange but prevent contaminations. Jars contained protist pellet medium (Carolina Biological Supplies), at a concentration of 0.55 g per liter of Chalkley’s medium and two wheat seeds for slow nutrient release. In addition, the medium contained three bacterial species (*Serratia fonticola*, *Brevibacillus brevis* and *Bacillus subtilis*) as food source for the ciliates. Jars were kept in a temperature-controlled incubator at 15° Celsius. Stocks were transferred monthly by pipetting a small subsample of the previous culture into a jar prepared as described above. Because the different ciliates used show quite pronounced intrinsic differences in cell density under the same culture conditions [17], variable numbers of trajectories were obtained per species.

### Data collection

Sampling was done on two dates (24.03.2014 and 07.04.2014) with cultures being 20 days old and thus in the stationary phase. We collected movement trajectories by videoing subsamples of the cultures. To do so, we transferred 1 ml of ciliate culture into a Sedgewick Rafter counting chamber, which was placed under the objective of a stereomicroscope (Leica M205 C) at 25x magnification. We took 20 second video sequences at a frame rate of 25 frames per second using a mounted digital CMOS camera (Hamamatsu C11440) resulting in a total of 500 frames. Dark field illumination was used such that ciliates, transparent in bright field microscopy, appear white on black background; this greatly facilitates the segmentation of videos. We used the software BEMOVI to extract morphological features and movement trajectories of individual cells [23]. Six morphological attributes were extracted for each fix: grey value (pixel intensity from 0 [black] to 255 [white]), area (i.e., cross section), the perimeter, major and minor axes of a fitted ellipse and the aspect ratio (i.e. minor axis/major axis [AR]). Trajectories were filtered by a standardized procedure to get rid of spurious trajectories due to swimming debris: trajectories for analysis were required to show a minimum net displacement of at least 50 pixel, 10 fixes per trajectory and a detection rate of 80% (i.e. a trajectory with a duration of 10 frames has to have at least 8 fixes) and a median step length of greater than 2 pixels. This resulted in 3957 trajectories in total.

### Analysis

Different movement features sets were first tested to assess their predictive power for finding species classes. These include all combinations of aggregate movement parameter (MP), approximate entropy (ApEn) and wavelet (Wav) features, leading to 7 movement models including: MP, ApEn, Wav, MP+ApEn, MP+Wav, ApEn+Wav, MP+ApEn+Wav. According to their performance, selected movement feature sets are later integrated to morphological features. The feature sets selected for this study (after initial performance evaluation) and the numbers of features are listed below:

1. *Morphology*: Mean and standard deviation for the 6 morphological attributes along the trajectory (12 features per trajectory)
2. *MP only*: Mean, standard deviation and median values for 7 movement parameters, i.e. distance travelled, speed, acceleration, turning angle, angular velocity, meandering and sinuosity (21 features per trajectory)
3. *MP+ApEn*: Adding ApEn values for all the movement parameter profiles to the MP model (7 additional features per trajectory; total of 28 [= 21+7]).
4. *MP+ApEn+Wav*: Adding wavelet features using profiles of the distance travelled (30 additional features; total of 58 [= 28+30])
5. *MP+Morph*: Integrates 21 *MP only* features and 12 morphology features (total of 33 [= 21+12])
6. *MP+ApEn+Wav+Morph*: Integrates all features, i.e. morphology and all features based on movement (total of 70 [= 58+12])

Since the number of the features notably increases, a feature selection process was employed to determine the ultimately relevant features in the classification. An evolutionary feature selection process by Genetic Algorithms (GA) in conjunction with the classifier (i.e. DT and SVM) was used to evaluate the significance of the added features in the classification. For SVM, we applied a radial basis function (RBF) with two kernel parameters of *C* = 20, which is a penalty parameter imposing a tradeoff between training error and generalization performance of SVM classifier and γ = 0.001, which is an exponent factor in the RBF function. In case of DT, a top-down procedure is applied based on the CART learner to traverse the tree, using the following parameter setting: maximal depth of tree = 20, minimal size for split = 4 and confidence value of 0.25. The reported results are based on a 10-fold cross-validation for both classifiers in the feature selection process, with the following parameter settings for GA: population size: 10, number of generations = 30, probability of cross-over = 0.5 and probability of mutation = 1 / (number of features).

For the evaluation of the performance of classification models, the overall classification accuracy and the Kappa coefficient are used. Kappa values are helpful when there is an imbalance in the number of instances between the classes [42], which is the case in our dataset. In case of individual species classes, precision and recall values were measured. Precision is calculated as True Positive / (True Positive + False Positive), whereas recall is defined as True Positive / (True Positive + False Negative).

## Results

### Contrasting morphology and movement features

**Fig 2:**
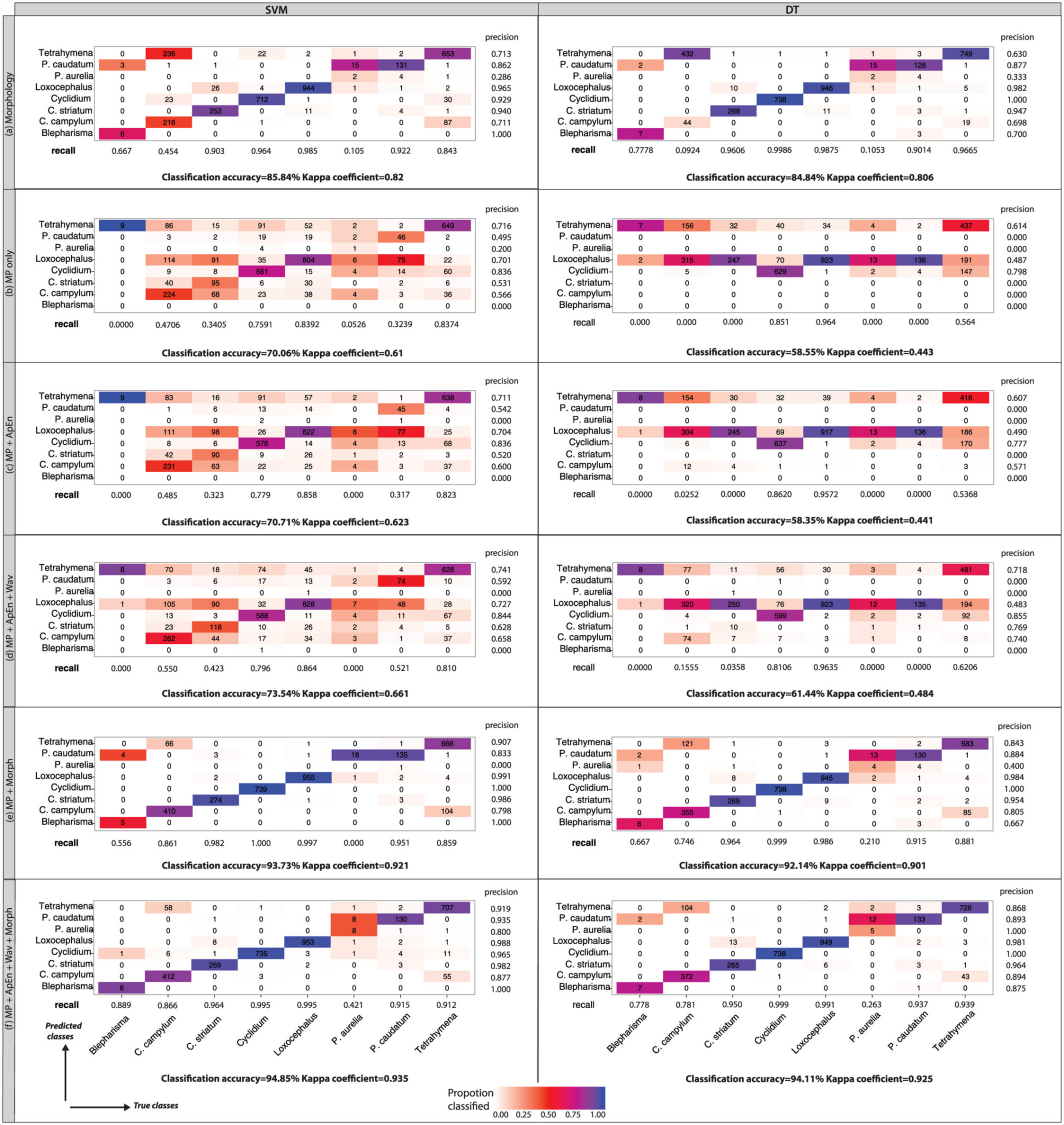
Confusion matrices obtained from SVM (left) and DT (right) by using different feature sets (sections a-f). The classification precision and recall values are shown for each class in all the tables. The cells are colored in order to indicate the classification precision for each class. Overall classification accuracy and Kappa values are shown below each confusion matrix. Although SVM generally outperforms DT, once both movement and morphology features are integrated, the results are very much comparable (section e and f).

The individual confusion matrices shown in Fig 2, as well as the overall accuracy and kappa values for different models (shown below the confusion matrices), allow to contrast movement and morphology features. The baseline *Morphology* model is quite successful in classifying most of the species, except for *Blepharisma*, *C*. *campylum* and *P*. *aurelia* which have low recall and precision values in both SVM and DT cases (Fig 2a). Overall, the *Morphology* model based on SVM reaches a classification accuracy of 86% and Kappa value of 0.82, which is comparable to the result of the decision tree with an accuracy of 85% and Kappa of 0.81 (Fig 2a). In contrast, classification accuracy based on the MP only model had a considerably lower accuracy of 70% and Kappa value of 0.61 (SVM), and 59% and Kappa of 0.44 (DT) than the baseline morphology only model (Fig 2b). Adding ApEn features led to a small increase using SVM, whereas classification accuracy of the DT slightly decreased (Fig 2c). Further adding wavelet features led to further classification improvement for both classification methods (Fig 2d). The combination of simple aggregate movement features and morphology improved the accuracy by 8% for both classifiers (Kappa 0.92 and 0.9 for SVM and DT, respectively) compared to the morphology only baseline. (Fig 2e). Importantly, the final classification model, which integrates both morphological and all movement features, resulted in similar performances of both classifiers: 95% classification accuracy and Kappa of 0.94 in case of SVM, and 94% accuracy and Kappa of 0.93 for DT (Fig 2f). Whereas the increase due to wavelet and ApEn features in addition to simple MPs looks small with only about 1–2% overall, species-specific improvements (especially for underrepresented species like *Blepharisma* and *P*. *aurelia*) in accuracy and recall may justify the inclusion of advanced features such as wavelets (Fig 2e and 2f). Although DT performed generally less well than SVM for all movement-based features, once morphological features were integrated it performed as well as SVM. Although the two classification methods used different numbers of features (29 vs. 43 for SVM and DT, respectively) to reach such similar classification success, individual features from all feature sets were used in both cases, highlighting the complementary information content in each feature set (Table 1).

**Table 1:** Number of selected features using SVM and DT in the final classification stage.

	Feature sets				
Models	MP (out of 21)	ApEn (out of 7)	Wavelet (out of 30)	Morphology (out of 12)	Total (out of 70)
SVM	11	3	9	6	29
DT	10	5	20	8	43

**Fig 3:**
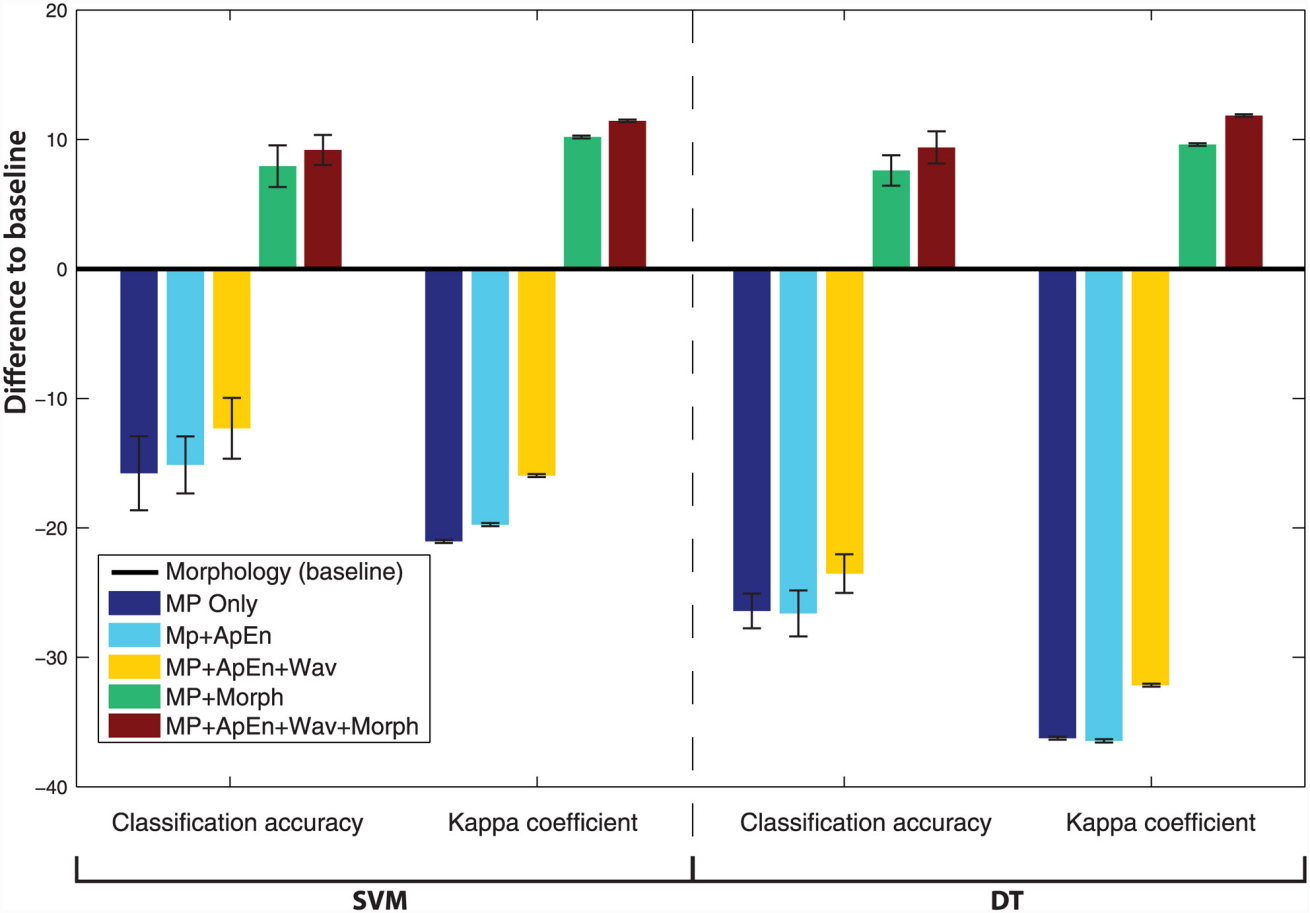
Comparison of the overall classification accuracy and Kappa coefficient using SVM and DT. Kappa values are scaled from [0–1] to [0–100], in order to make them comparable with accuracy values. The morphological model is considered as the baseline (0 on the Y axis) and the deviation of models using different feature sets are compared (model—baseline). The error bars shown for each bar plot are derived from the different folds of the cross validation and assist to judge the significance of the increase. Classification based on movement features fares less well than morphology alone, but once integrated, movement features increase both the classification accuracy and Kappa coefficient by about 10%.

In order to compare the performance of different models to the morphology baseline, we looked at the difference in the overall classification accuracy and Kappa values between particular movement feature sets and morphology (Fig 3). The movement-based features, on their own, were inferior in both accuracy and Kappa compared to the baseline. However, there is an improvement compared to the baseline once complementary features sets are added to the classification model. When morphology and all movement features were integrated, the reported classification accuracy (9%) and Kappa coefficients (0.12) improved substantially, for both SVM and DT, compared to morphology only.

### Species classification based on movement features alone

**Fig 4:**
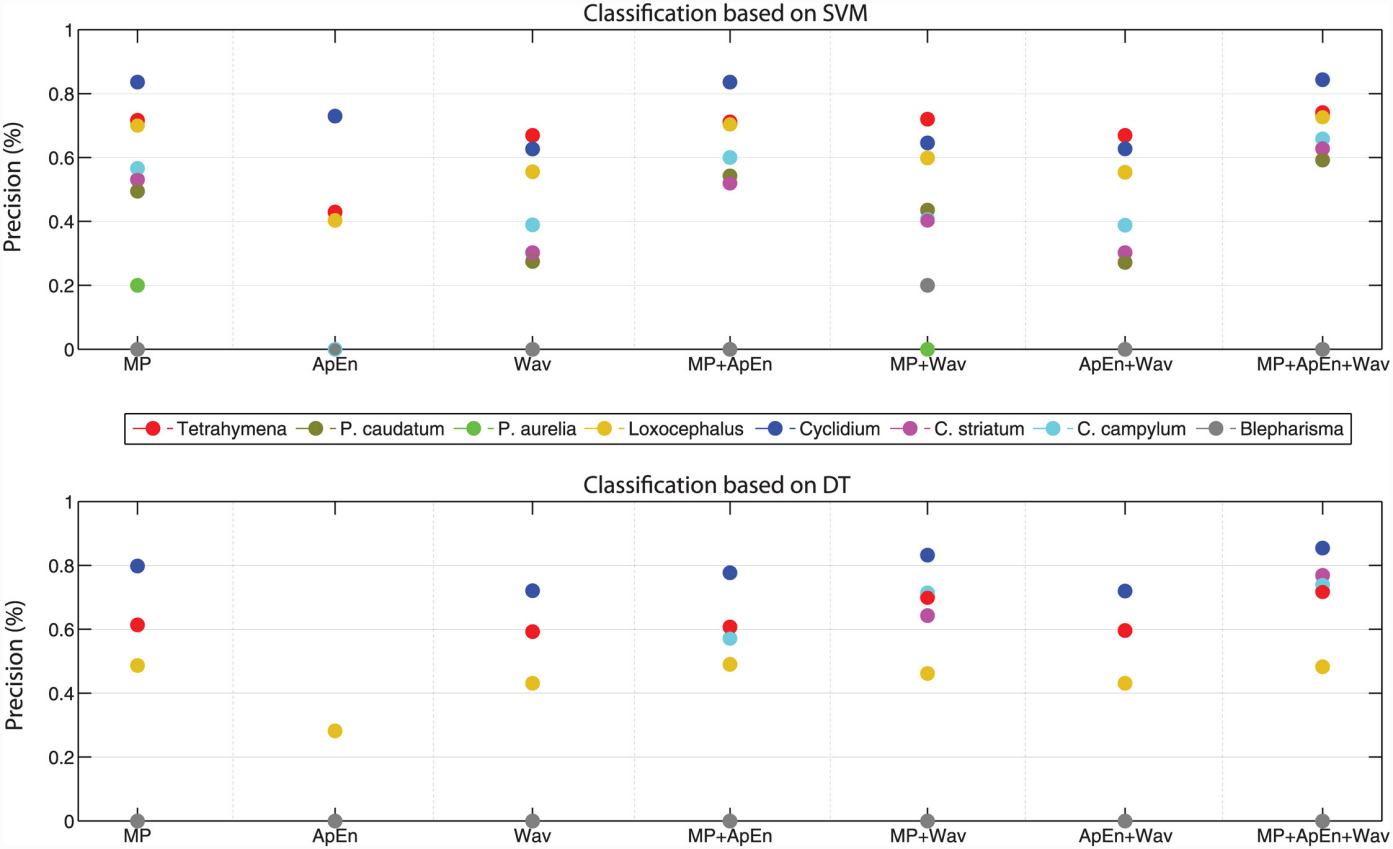
Precision values of predicted ciliate species based on different movement feature sets. MP is based on 21 general movement features, ApEn are the 7 ApEn features and Wav is based on 30 wavelet features. For the overlapping cases (e.g. when the precision values are zero), only the dots for one class (e.g. *Blepharisma* in grey) are shown.

As classification of ciliate species based on movement features is relatively uncommon in the literature, we here compare the models based on movement features only, which would be useful if no information on morphology is available, or the morphology information is unreliable. Classification based on these sets of movement features revealed that certain features perform better than others when classifying species (Fig 4). Unsurprisingly, ApEn and the wavelet features alone were less successful in predicting species compared to the MP features, as they only characterize specific aspects of movement. However, once integrated with MP features, they increased the classification performance in almost all cases. Three species (i.e., *Cyclidium*, *Tetrahymena* and *Loxocephalus*) were quite well predicted by movement features alone, regardless of the classification method, whereas two classes (i.e., *Blepharisma* and *P*. *aurelia*) failed to be correctly predicted by any of the movement feature sets alone (Fig 4). Advanced movement features seem most important for *Colpidium striatum* and *C*. *campylum*, although the performance increase is only subtle when only movement features are considered. However, overall there is a steady improvement in the performance of both classifiers when movement features are added.

## Discussion

Our results demonstrate that: 1) although classification models based only on movement features do not perform as well as morphological features, the integration of both feature sets results in better classification performance than each set alone; 2) adding movement features that are complementary to simple MPs aggregated on the trajectory level increases the classification success overall only slightly, although their contribution can be important for particular classes (especially underrepresented species in the case study here) and may vary with the classifier used; 3) once feature sets are integrated, performance of different classification methods (i.e. SVM or DT) are comparable and allow accurate and robust classification.

Classification performance based on the *MP only* model is comparable to classification based on morphology in other studies. For instance, a study looking at the automated classification of cichlid fish from Lake Malawi classified on average about 78% correctly [43], whereas another study comparing functional groups of plankton classified about 82% correctly [44]. It should be noted, however, that the numbers of classes were higher (12 combinations of species and sex in the former and 53 functional groups in the latter) in these two studies than in our case. Another study aiming for classification of 6 different movement classes of fish (i.e. a lower number as in our case) had an accuracy of 74% [45], comparable to our results based on movement only. This shows that movement features on their own, are a worse proxy than morphology for species classification in our particular case, but may still provide a worthwhile information gain in other systems, especially when automated classification outperforms human observers [43]. A possible explanation for movement being a less good predictor on its own, is the inherent variability of movement compared to morphology, which may only vary in the restricted range of morphological development. It is known for instance that phenotypic plasticity is larger for behavioral traits (which would include movement) than for instance morphological traits, as shown in a study by [46]. What would be fruitful avenues to improve species classification based on movement behavior? The temporal scale covered by our case study (20 seconds) is still relatively small compared to the lifetime of a cell (several hours to a day) and potentially the temporal scale of behavior. Hence, if we capture only a fraction of the actual behavioral mode, it may be difficult to characterize the species with that information because species identity and behavioral mode may be confounded. A study looking at the movement behavior of cows has shown that the temporal resolution and length of the trajectory determined whether behaviors could be reliably detected or not [47]. Increasing the overall length of the trajectory may help in better capturing the characteristic features of ciliate movement and hence its classification. In addition, the frequency and composition of behavioral modes expressed during the lifetime of a cell may have higher predictive power regarding species identity, as species may show specific signatures of behavioral modes when compared to each other. It was, for instance, shown that movement behavior does vary over the lifetime of cells, although most of the variation can still be summarized in two major behavioral modes [48]. It has to be noted, however, that longer videos have increasing demands in processing power and storage, which may only be justified when higher resolution in terms of behavioral modes is desired and classification success has to exceed the already high success rate shown in our study.

Slight classification performance improvements when features based on ApEn were added to the *MP only* model might be due to the fact that in the dataset used, dominant fluctuations or regularities are not really present, or that these are similar among classes. The movement of different ciliate species is rather similar to each other (as can be seen in the results of the *MP only* model) and detecting any dominant regularity in the MP values (captured through ApEn) is rather difficult in our case. One reason for the strongly converging movement behavior among species may be the shared foraging mode. The 8 ciliate species used are all bacterivorous species feeding by phagocytosis, i.e. the engulfing of food particles such as bacteria during swimming [49]. Because they share similar bacterial prey, natural selection may have led to the evolution of very similar movement strategies that allow similar foraging success among species. Although the ApEn features were not largely contributing to classification success, they still yielded a slight improvement to the classification in the case of SVM. Thus, we retained ApEn features in the classification to test if they were considered in the final feature selection process.

The third classification model including wavelet features further improved classification success. This shows that the wavelet features have been successful in capturing periodic movements in ciliate trajectories. These periodic patterns in at least some of the ciliate species could, for instance, be due to a looping behavior, where individuals move away from their departure point and return within a given time period [50]. Such a movement pattern would lead to periodic changes in the net displacement. It is most likely that these movements are performed on a small spatial scale such that they were captured by the wavelet analysis. In other applications such as the classification of EEG signals, wavelet analysis has been successfully applied, owing precisely to the periodic nature of the signals [41,51]. Our study shows that wavelet analysis provides complementary information to static movement parameters and hence improves classification success by capturing an additional aspect of movement. Importantly, adding the complementary wavelets and ApEn also improved the overall classification success from 89% using static movement parameters and morphology to 95% in this study [23]. However, as shown in Fig 4 wavelet or ApEn features on their own are less meaningful in movement based classification problems, since they will only capture specific aspects of movement such as periodic patterns.

Contrasting the morphology + MP model with the morphology + MP + advanced movement features model shows that the advanced features have merit in terms of improving species-specific accuracy and recall. Both species with the lowest number of cases (*Blepharisma* and *P*. *aurelia*) had improved accuracy and recall and even the abundant *Tetrahymena* was better classified. The increased effort of calculating advanced movement features hence pays off due to the improvements, but simple movement metrics may be preferred if the movement expressed does not show temporal structure (as for other species such as *Colpidium*, *Cyclidium* and *Loxocephalus*). Interestingly, the advanced movement features contribute only to improved classification in the case of these species, when combined with morphology, as classification only based on movement failed completely. This suggests that combined features can have synergistic effects on classification performance and the right combination of features is key for a successful overall classification.

Another achievement of this study is demonstrated by the results of the final classification model that integrates all the movement and morphology features: Careful selection of input features to obtain a set of features that collectively capture the varied aspects of movement will result in the highest classification performance, regardless of the classification method used. In this study, two classification methods with different theoretical background were employed (i.e. SVM and DT). While we were not comparing the performance of those methods, we would like to point out that selecting relevant movement features capturing different aspects of movement is of utmost importance. Such a classification approach can ensure reliable results, as can be seen through the comparable sets of selected features for building the SVM and DT models. This also could be seen in the range of selected features shown in Table 1, where SVM achieves similar results with fewer features. In the *MP only* model, all the movement parameters are used for both DT and SVM models. In the case of ApEn, SVM uses only 3 features (ApEn of distance travelled, acceleration and turning angle), compared to 5 used by DT (distance travelled, acceleration, speed, meandering and sinuosity). The selected features based on the wavelet transform show that features corresponding to different approximations and sub-bands are intermittently used, confirming the importance of both of these sub-bands in the classification. Although the two sets of selected features for SVM and DT are not exactly the same, all the developed groups of movement features showed up in the feature selection process, indicating their contribution to classifying between species. This is in accordance with the findings of other studies [26], where different combinations of features may end up in comparable results. The final message is that the combination of relevant features—movement and morphology in our case study—can ultimately build reliable classification models with high precision and recall.

Previous classification based on random forest classification showed that imbalance in the abundance of classes would influence the outcome for specific pairs of species. For instance, *P*. *aurelia* being less abundant than *P*. *caudatum* would get completely lumped into *P*. *caudatum* [23]. Whereas not unexpected due to the working principle of the random forest algorithm, the classification is unreliable for the minority class. Here we show that other classification methods such as SVM can accommodate for such imbalances better and may therefore be better suited when dealing with datasets that show large imbalances as the one used in this study.

We also employed the approach presented in [26], where a moving window of different (temporal) sizes is employed for the computation of MPs and then imported to the classification model. The results of this simple cross-scale analysis method, although not presented here, suggested that the original temporal granularity at which the data was captured was the most reliable temporal scale for the calculation of MPs. Consequently, when we employed wavelet analysis, we saw that adding features based on the DWT indeed contributed to improving the performance of the classification. We conclude from this that since scale issues manifest themselves in different ways in movement analysis, appropriate methods need to be used in order to provide complementary measures to scale-specific techniques.

As part of future work, the capability of the discrete wavelet transform will be investigated in other relevant problems in movement research, including trajectory segmentation. Movement classification and segmentation share common characteristics, given that they both aim at grouping parts of trajectories with respect to the similarity in movement properties. Due to similar conceptual backgrounds, the features extracted from movement trajectories can be used towards both classification and segmentation. Hence, features developed for the classification of entire trajectories could also be applied to subtrajectories, with little modification. The focus in the case of segmentation is to divide the trajectories into segments (subtrajectories) with homogeneous movement characteristics, which can point out the particular behaviors to be mined from movement trajectories. Since DWT decomposes the input signals at different levels, it can be used to investigate the variation of behaviors across different scales. This can be particularly interesting in different application domains, where sophisticated methods are needed to automate the process of segmenting large volumes of movement data.

## Conclusions

In this study, the contribution of different movement features in a classification problem was investigated. Different ciliate species were considered as the target classes, to assess whether features based on movement can be employed as a complementary proxy to morphology in the classification problem. Our results demonstrate the value of exploring wavelet analysis, together with general movement features, in order to better distinguish the ciliate species. Such features have not been used yet in studies related to automated classification of species in the context of video analysis, and are so far rarely employed for feature extraction in movement classification studies in general. We believe that our findings are applicable to movement ecology studies in general, since they show that movement paths can be automatically classified according to classes such as species, but may also be useful to infer biological states such as behavioral modes. Our results also have potential application for instance in the field of automated monitoring of waste water.

## Supporting Information

S1 Fig Working principle of the discrete wavelet transform. (EPS)

S2 Fig Decomposition of the movement parameter profile through wavelet analysis at different levels. (EPS)

S1 Text Movement classification by the discrete wavelet transform. (DOCX)
