# Correction: Developing and Integrating Advanced Movement Features Improves Automated Classification of Ciliate Species

**DOI:** 10.1371/journal.pone.0148592

**Published:** 2016-01-29

**Authors:** The PLOS ONE Staff

The Data Availability statement for this paper is incorrect. The correct statement is: Data have been deposited to Figshare: http://dx.doi.org/10.6084/m9.figshare.1619874. The publisher apologizes for the error.
