# Supplementary material for: Developing and Integrating Advanced Movement Features Improves Automated Classification of Ciliate Species
Source: PLoS One. 2015 Dec 17;10(12):e0145345. doi: 10.1371/journal.pone.0145345 (PMC4682988; doi:10.1371/journal.pone.0145345)
Supplement: S1 Text — (DOCX) [file pone.0145345.s003.docx]

## S1 Text. Movement classification by the discrete wavelet transform

To our knowledge, the discrete wavelet transform (DWT) has rarely been used in movement analysis, but has been successfully applied in classification problems such as the context of EEG signal classification [1,2] or image compression [3,4]. One ecological example is the work by [5], where change points were detected to segment animal movement trajectories. The value of the wavelet transform lies in the decomposition of the original movement parameter profile into multiple levels allowing the quantification of periodic characteristics of movement across different scales.

### Overview of the wavelet transform

Wavelet analysis is a time-frequency representation of a signal, providing a better time and frequency resolution compared to the short-time Fourier transform (STFT). By varying the time-frequency aspect ratio, good frequency localization at low frequencies (i.e. long time windows), and good time localization at high frequencies (i.e. short time windows) are achieved [3,6]. The wavelet transform (WT) is the process of expressing an input signal through a set of functions, by shifting and dilating a single function called the mother wavelet function. This makes it possible to decompose the input signal at different scales, leading to a set of coefficients at each level called wavelet coefficients.

Two forms of WT are possible: the continuous wavelet transform (CWT) and the discrete wavelet transform (DWT). For our purpose of generating input features for a classification model, the CWT will generate an excessive amount of data, which cannot easily be aggregated and fed into the classification model. In contrast, the DWT is similarly accurate as the CWT [3] and further analysis of the wavelet coefficients is done by summarizing the coefficients of different decomposition levels as input features for the classification. In the following, we will focus exclusively on the DWT.

Using the DWT, the signal *S* is successively decomposed into multiple levels of resolution (S1 Fig). This decomposition procedure yields multiple approximations and detail information by passing through low-pass and high-pass filters, respectively. The low-pass filter keeps only the frequencies lower than a certain threshold, leading to maintain the general structure of the signal. Whereas high-pass filter allows passing signals with frequencies higher than the threshold, allowing to capture details of variation in the signal. The decomposition involves subsampling of the original signal at dyadic scales, *x* = 2*^j^* (with *j* = 1, 2, …, *k* levels). The original signal then can be calculated as the summation of approximations *A_j_* and details *D_j_*.

S1 Fig. Working principle of the discrete wavelet transform. *S* *=* *A_j_ + j * D_j_*, where *A_j_* represents the approximation at the final level of decomposition and *D_j_* represents the corresponding detail. *h[n]* is the high-pass filter and *l[n]* is the low-pass filter.

Summarizing the extracted information is required to use it as input features in the classification. In the discrete wavelet transform, this may be accomplished by summarizing the wavelet coefficients.

### Application of the discrete wavelet transform to ciliate movement trajectories

In the feature extraction step, all the approximations *A_1_* .. *An* and the details *D_1_* .. *D_n_* of the MP signals were considered. The importance of the approximation sub-bands lead us to integrate all the features based on them. The approximation component captures the general structure of the signal and the detail component is able to capture the variations in the signal. These two sets of information are adequate to reconstruct any input signal [3,6]. Therefore, in most of the studies using the DWT, the moment statistics of these components are used as the input features [2]. However, the selection of the sub-bands from which the features are extracted depends on the patterns that are to be mined from the data. For example, in [5], the main interest was to pinpoint the change points in the movement and this information lies in the high-frequency component of the signal, hence the required information could be obtained by merely looking at the sub-bands. However, in our classification problem the behavioral patterns that form the movement classes manifest themselves in intervals that cover a certain time period and thus affect the general structure of the signal. Therefore, we used the approximation together with the detail components in order to investigate the movement classes.

Three summary statistics were computed for each sub-band and considered as input features for the classification. These include the mean of the absolute values of the coefficients in each sub-band; average power of the wavelet coefficients in each sub-band; and standard deviation of the coefficients in each sub-band.

This approach also helped in selecting the appropriate number of decomposition levels in the wavelet analysis, since it is an important factor in the analysis of signals using the DWT. In the literature, the number of decomposition levels has been decided based on the dominant frequency components of the signal, such that those parts of the signal that correlate well with the frequencies necessary for classification of the signal are retained in the wavelet coefficients [2]. According to [5], the DWT eliminates noise at the 1st and 2nd level of decomposition and the discontinuity in the signal becomes visible at the 4th or 5th decomposition. Our findings are in agreement with these observations, as most of the information needed for the reconstruction of the signal is retained quite well up to the 5th level of decomposition (S2 Fig).

S2 Fig. Decomposition of the movement parameter profile through wavelet analysis at different levels. The approximation and detail sub-bands as well as the obtained wavelet coefficients are shown for each level.

For the mother wavelet function, as another deciding factor in the wavelet analysis, different functions were assessed in order to determine their effect on the classification performance. A Daubechies wavelet was chosen, due to its superior performance, and order 4 selected to detect the discontinuities in the signals.

Among the MP profiles employed in this study, the distance travelled and speed are the most commonly used parameters in conjunction with the wavelet analysis in the literature [5,7,8]. For both, periodic patterns in the profile may be expected whereas for other parameters, it would be rather difficult to interpret the meaningfulness of periodic patterns in their profiles. Classification experiments that we conducted solely using wavelet-based features supported the findings from the literature: superior results could only be obtained using one of these two parameters. In the experiments reported here, we used the profiles of the distance travelled to extract the wavelet-based features.

## References

1. Güler I, Ubeyli ED. Adaptive neuro-fuzzy inference system for classification of EEG signals using wavelet coefficients. J Neurosci Methods. 2005 Oct 30;148(2):113–21.

2. Subasi a. EEG signal classification using wavelet feature extraction and a mixture of expert model. Expert Syst Appl. 2007 May 22;32(4):1084–93.

3. Mallat SG. Multifrequency channel decompositions of images and wavelet models. IEEE Trans Acoust. 1989;37(12):2091–110.

4. Li J, Gray RM. Context-based multiscale classification of document images using wavelet coefficient distributions. IEEE Trans image Process. 2000 Jan;9(9):1604–16.

5. Sur M, Skidmore AK, Exo K-M, Wang T, J. Ens B, Toxopeus a. G. Change detection in animal movement using discrete wavelet analysis. Ecol Inform. Elsevier B.V.; 2014 Mar 5;20:47–57.

6. Daubechies I. The wavelet transform, time-frequency localization and signal analysis. IEEE Trans Inf Theory. 1990;36(5):961–1005.

7. Wittemyer G, Polansky L, Douglas-hamilton I, Getz WM. Disentangling the effects of forage, social rank, and risk on movement autocorrelation of elephants using Fourier and wavelet analyses. Proc Natl Acad Sci. 2008;105(49):19108–13.

8. Riotte-Lambert L, Benhamou S, Chamaillé-Jammes S. Periodicity analysis of movement recursions. J Theor Biol. 2013 Jan 21;317:238–43.
